# Associated factors, triggers and long-term outcome in Complex Regional Pain Syndrome (CRPS) in the upper limb – A descriptive cross-sectional study

**DOI:** 10.1371/journal.pone.0320263

**Published:** 2025-03-28

**Authors:** Astrid Parinder, Ellen Lyckegård Finn, Lars B. Dahlin, Erika Nyman

**Affiliations:** 1 Department of Translational Medicine – Hand Surgery, Lund University, Malmö, Sweden; 2 Department of Hand Surgery, Skåne University Hospital, Malmö, Sweden; 3 Department of Biomedical and Clinical Sciences, Linköping University, Linköping, Sweden; 4 Department of Hand Surgery, Plastic Surgery and Burns, Linköping University Hospital, Linköping, Sweden; Southern Medical University Nanfang Hospital, CHINA

## Abstract

The pathophysiology behind Complex Regional Pain Syndrome (CRPS) is not fully understood and associated factors and triggers for developing the condition are debated. We aimed to study such factors and long-term outcome in a descriptive cross-sectional study with a well-defined population with CRPS in the upper limb and related to sex and CRPS type. In retrospectively collected data from medical records, 149 subjects [women n = 104 (70%); type 1 CRPS, n = 108 (72%); type 2 CRPS, n = 41 (28%); follow-up time 21 [8-43] months] were identified and analysed (Chi-squared test, Mann-Whitney U-test, and multiple linear regression). A majority were manual workers, and a larger proportion of subjects were smokers and had less post-secondary education than a reference population (p < 0.001 and p < 0.008). Men were younger, more frequently smoked, had higher BMI, and had lower education levels than women (p = 0.044, p = 0.007, p < 0.001, and p = 0.016, respectively). Subjects with CRPS type 2 were younger and had a longer time from symptoms until diagnosis, longer follow-up time, and more follow-up visits, indicating worse outcome (p = 0.016, p = 0.0012, p = 0.003, and p = 0.004, respectively). Among CRPS, 32% had a prior pain disorder and 7% had previously visited a pain management clinic. While there was no significant difference in mental illness occurrence before CRPS diagnosis compared to a reference population, mental illness increased by 76% after diagnosis. Factors such as CRPS type 2, older age, and delayed diagnosis were associated with longer follow-up periods. Additionally, 45% were on sick leave for over 12 months, and 20% were permanently unable to work. Socioeconomic deprivation is an associated factor in developing CRPS, in which a variety of triggers exist. Subjects with CRPS, particularly type 2, are at high risk of severe remaining symptoms, including mental illness and risk of never returning to work.

## Introduction

Complex Regional Pain Syndrome (CRPS) is a long-lasting severe chronic primary pain disorder, now updated in ICD-11 and likely a nociplastic pain, often induced by trauma, surgery, or immobilization [1–5]. The process tends to start distally affecting both upper and lower limbs with a reported distribution of 3:1 [6,7]. The diagnosis is based on the internationally agreed Budapest criteria, including exclusion of other potentially interfering disorders and assessing clinical criteria [8–10]. CRPS is characterized by pain (nociplastic pain; > 3 months duration, regional rather than discrete distribution; pain not entirely explained by nociceptive or neuropathic pain mechanisms) disproportionate to the possible previous events or trauma and has a high frequency of allodynia and hyperalgesia, accompanied by a wide variety of autonomic and motor disturbances [8,9]. The incidence of CRPS is reported to be 13.6 per 100,000 per year [11], but it may have decreased as reported in the Netherlands [2,12–15].

CRPS is distinguished between type 1 and type 2, where an injury in at least one peripheral nerve has been identified in type 2 and no evident nerve injury in type 1 [8]. Although symptoms and available treatment options are similar, the aetiology and the initial pathophysiology motivate a distinction between the two types [8,16], but it is a matter of research [3].

None of the reported pathophysiological theories, also including highlighted disturbances in the motor and somatosensory cortex, can fully explain the complex and different symptomatic patterns of CRPS [3,7,17–20]. CRPS is more prevalent in women [11,17,21], with reported associated comorbidities [22,23] that differ from those in chronic musculoskeletal pain [24]. The psychological behaviour, depression, and preoperative psychological distress or increased pain level are also discussed in the context of predictive or associated factors for the development of CRPS as well as for prognosis [25–30], which is interesting given a described bidirectional relationship between pain and mental illness [31]; thus, a biopsychosocial approach in the management of the disorder [3]. Neuroinflammation and neuro-autoimmunity are also highlighted and with an association to pain that may not follow the distribution of nerves or dermatomes as well as to unusual movement disorders, and somato-visceral dysfunctions [32]. Among the subjects who get CRPS after being diagnosed with carpal tunnel syndrome (CTS), 50% demonstrate normal neurophysiology prior to surgery [33], which is unusually high as related to the reported sensitivity and specificity of neurophysiology [34]. Thus, one may ask if subjects with CTS are correctly diagnosed or if their symptoms may signify a condition of imminent CRPS [33]. As potential risks or associated factors are still vaguely defined, it is currently impossible to prospectively foresee which subjects will become affected [35], since there are no single biomarkers available for diagnosis [23]. Hence, to better predict CRPS and to optimize diagnosis and treatments, a deeper understanding of potential associated factors or triggers and knowledge of long-term outcome is needed [1,2,7–9].

In a descriptive cross-sectional study, with included retrospective data, we aimed to identify associated factors and triggers for developing CRPS in the upper limb as well as to study the selection of treatments and long-term outcome in subjects diagnosed with CRPS in a well-defined region and related to sex and type of CRPS.

## Materials and methods

### Data collection procedures

#### Patient population.

**Fig 1:**
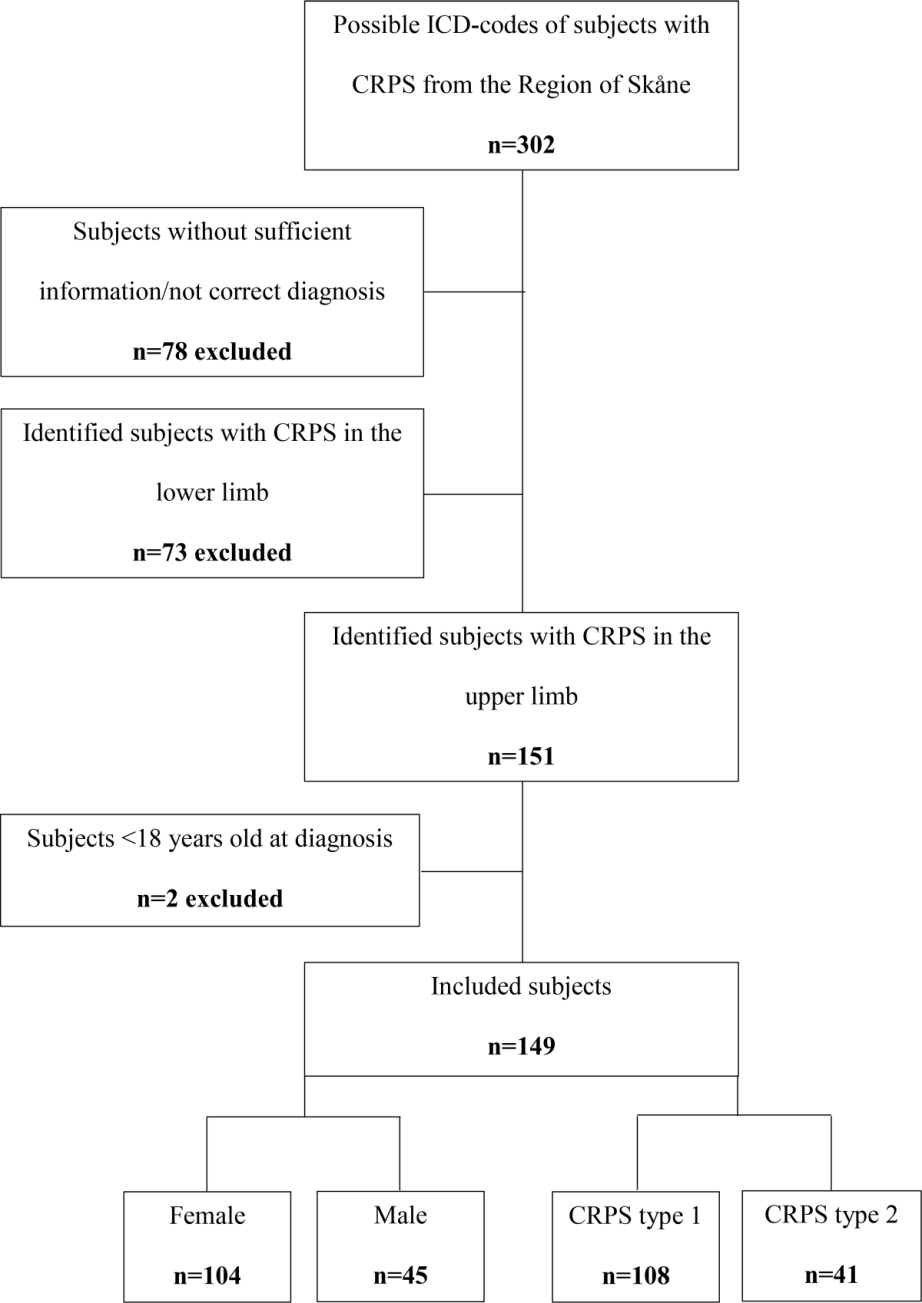
Flow chart illustrating identified and included subjects with Complex Regional Pain Syndrome (CRPS) in the upper limb divided by sex and type of CRPS.

Subjects, at the age of ≥ 18 years, diagnosed with a documented and well-defined CRPS or algodystrophy (defined and based on the ICD-10 codes G90.5, G90.6, G90.7, M89.0) from 1 January 2007 to 31 December 2021, and treated at Department of Hand Surgery, Department of Orthopaedics and at the Department of Pain Rehabilitation, in the Region of Skåne, Sweden were identified and included. Given that CRPS can present differently in the upper and lower limbs, only subjects with CRPS in the upper limb were included in this study. Subjects with pain conditions lacking the correct diagnosis code, symptoms not matching the Budapest criteria [9,15], or with insufficient information in the medical records were excluded. A total of 302 subjects were initially identified of which finally 149 subjects were included in the analysis (Fig 1). Data from the medical records of the included subjects, including previous referral requests and surgery reports, were compiled based on a list of variables, focusing on associated factors, triggers and outcome, and retrospectively analysed with focus on sex and type of CRPS. In addition, the different words for the variables were used as search terms in the medical records. The two CRPS types were determined based on symptoms and triggers of CRPS. No informed consent was obtained as approved by the Ethical Review Authority. Data were accessed for research between 14 February 2022 and 14 May 2022.

#### Analysed variables – associated factors, triggers, and outcome.

A variety of symptoms and *associated factors* were evaluated (e.g., smoking, dominant hand, civil status, profession, BMI, duration of symptoms until diagnosis, previous surgery, history of pain or CRPS, level of education, and co-morbidities). If not mentioned in the medical records, education level was determined based on the expected degree of education needed for current or previous employment. Mental illness was based on the information in the medical records about treated conditions, like depression, anxiety, and PTSD, but no severe psychiatric conditions were present (e.g., schizophrenia). The presence of neuropathic pain, including symptoms described by the subjects like sensation of burning, stabbing, and/or radiating pain, was evaluated from the medical records [36].

All subjects whose *trigger* was surgery for CTS, as well as other nerve release surgeries, were included as CRPS type 2 [6,15]. The category of trauma as a trigger included fractures, ligament ruptures, wounds, electric shock, joint dislocation, biceps tendon rupture, and other types of violence against the limb.

Different methods concerning clinical investigations and selected treatments were defined. *Outcome* was defined with different criteria, e.g., as follow-ups (number of surgery, outpatient and rehabilitation visits, department for visit, follow-up time), time of sick leave, back to work, or permanent incapable of work. Numbers of follow-up visits to physicians and physiotherapists/occupational therapists were counted from accessible patient folders from hospitals in Region Skåne, meaning that subjects treated in the outpatient care setting or at a private clinic may have had more visits. To make a comparison with the study population, data from multiple sources (for specific references, see the result section) were compiled to establish a reference population.

### Statistical methods

Categorical data are presented as numbers (valid %) and are analyzed using a Chi-squared test (or Fisher´s exact test if > 20% of expected cell counts were < 5). Data were not non-normally distributed, why they are presented as median [interquartile range; 25^th^-75^th^ percentiles], and analyses were made with Mann-Whitney U-test. A multiple linear regression was made to analyse any association between the dependent variable *follow-up time* as a proxy for outcome and the independent variables: sex, age at diagnosis, smoking, profession, duration of symptoms until diagnosis, previous surgery in the same hand, and neuropathic pain as symptom (unstandardized B [95% CI]; p < 0.05; with age and sex at diagnosis as adjusted factors). A p-value of < 0.05 was considered statistically significant. All statistical analyses were performed using the software SPSS (IBM corp. Released 2023. IBM SPSS Statistics for Windows, Version 29.0.2.0 Armonk, NY: IBM Corp).

### Ethics

The study was conducted according to guidelines, regulations, and national law as well as adhered to the principles in the Declaration of Helsinki. The study was approved by the Swedish Ethical Review Authority (https://etikprovningsmyndigheten.se/en/; permission no 2021-05570-01) based on the national Law for ethical review of research on humans. According to local regulations, permission was also obtained from the healthcare sector in Region Skåne, Sweden; so-called KVB-group for permission and decision, permission no 328-21) to access patient folders and use such data. According to the ethical permission (Swedish Ethical Review Authority; i.e., the same authority as above and permission no as above) and the KVB-decision (same KVB-group in the health care sector of Region Skåne, Sweden, same permission no as above), and based on the national Law for ethical review on humans, no informed consent is required from each subject for this type of research (i.e., data obtained from patient folders).

## Results

### Subject characteristics – sex and type of CRPS and associated factors

Among the 149 identified subjects [104 (70%) women, 45 (30%) men, 108 (72%) subjects with CRPS type 1, 41 (28%) subjects with CRPS type 2; Women: type 1 79 (76%) subjects, type 2 25 (24%) subjects; Men: type 1 29 (64%) subjects, type 2 16 (36%) subjects, p = 0.17] the median age at diagnosis was higher for women and those with CRPS type 1 (p = 0.044 and p = 0.016, respectively); median follow-up time since the end of treatment at the involved clinic (hand surgery, orthopaedic, pain management clinic) 21 [8-43] months. Based on the information from medical records (i.e., all specific Budapest criteria mentioned in records) 70/149 (47%) subjects had the defined criteria (no difference between females and males (p = 0.37) or between CRPS type 1 or type 2 (p = 1.00).

Men more frequently smoked and had a slightly higher median BMI than women (p = 0.007 and p < 0.001, respectively). Furthermore, no significant differences were found between sex or CRPS types regarding dominant hand, civil status, or profession (Table 1). A significant difference was seen between sex and education level as a higher proportion of women had a post-secondary education compared to men (p = 0.016).

**Table 1:** Characteristics of subjects diagnosed with Complex Regional Pain Syndrome (CRPS) in the entire cohort and split by sex and type of CRPS.

	Entire Study population (n = 149)	Female (n = 104)	Male (n = 45)	P-value	CRPS type 1 (n = 108)	CRPS type 2 (n = 41)	P-value
**Sex (female/male)**	104/45 (70/30)	NA	NA	NA	79/29 (73/27)	25/16 (61/39)	0.15
**Age at diagnosis (years)** ^a^	48 [36-58]	49 [38-59]	43 [30-53]	**0.044**	50 [38-60]	45 [33-51]	**0.016**
**Smoking (yes/no)** ^b^	48/92 (34/66)	27/72 (27/73)	21/20 (51/49)	**0.007**	35/64 (35/65)	13/28 (32/68)	0.85
**Body mass index** ^c^	26 [23–30]	26 [22–29]	28 [26–32]	** < 0.001**	26 [23–30]	27 [25–32]	0.12
**Dominant hand** ^d^				0.13			0.29
Right	95 (90)	71 (94)	24 (83)	–	68 (92)	27 (87)	–
Left	9 (9)	4 (5)	5 (17)	–	6 (8)	3 (10)	–
Ambidexter	1 (1)	1 (1)	0 (0)	–	0 (0)	1 (3)	–
**Civil status** ^e^				0.06			0.56
Married/Cohabited	98 (68)	74 (73)	24 (56)	–	72 (69)	26 (63)	–
Single household	47 (32)	28 (27)	19 (44)	–	32 (31)	15 (37)	–
**Profession**				0.54			0.15
Manual work	92 (61)	60 (58)	32 (71)	–	61 (56)	31 (76)	–
Non-manual work	34 (23)	26 (25)	8 (18)	–	27 (25)	7 (17)	–
Unemployed	1 (1)	1 (1)	0 (0)	–	1 (1)	0 (0)	–
Retired	16 (11)	13 (12)	3 (7)	–	15 (14)	1 (2)	–
Sickness compensation	6 (4)	4 (4)	2 (4)	–	4 (4)	2 (5)	–
**Level of education** ^f^				**0.016** ^g^			0.62
Elementary school	8 (6)	6 (7)	2 (5)	–	6 (7)	2(5)	–
Upper secondary school	90 (67)	54 (59)	36 (84)	–	60 (65)	30 (73)	–
Post-secondary education	36 (27)	31 (34)	5 (12)	–	27 (29)	9 (22)	–
**Duration of symptoms until diagnosis (weeks)** ^h^	15 [8-39]	14 [8-32]	16 [8-48]	0.74	12 [8-25]	24 [11-71]	**0.012**
**CRPS in dominant hand** ^i^				0.64			0.72
CRPS in dominant hand	68 (64)	50 (64)	18 (62)	–	46 (62)	22 (67)	–
CRPS in non-dominant hand	37 (35)	26 (33)	11 (38)	–	27(36)	10 (30)	–
CRPS bilateral	2 (2)	2 (3)	0 (0)	–	1 (2)	1 (3)	–
**Comorbidity (yes/no)**	108/41 (73/27)	78/26 (75/25)	30/15 (67/33)	0.30	76/32 (70/30)	32/9 (78/22)	0.35

Values are presented as number and proportion of observations (n (%)) or median (interquartile range; IQR]. P-values are based on Chi-squared test (or Fisher´s exact probability test if a group had n <  5) for categorical variables or Mann-Whitney U-test for numerical data. A p-value of < 0.05 was considered statistically significant and is indicated in bold.

^a^Due to unavailable information of year of diagnosis, 11 subjects are missing (8 females, 3 males/ 8 CRPS type 1, 3 CRPS type 2).

^b^Due to unavailable information of smoking, 9 subjects are missing (5 females, 4 males/ 9 CRPS type 1).

^c^Due to unavailable information of body mass index, 18 subjects are missing (10 females, 8 males/ 17 CRPS type 1, 1 CRPS type 2).

^d^Due to unavailable information of dominant hand, 42 subjects are missing (26 females, 16 males/ 34 CRPS type 1, 8 CRPS type 2).

^e^Due to unavailable information of civil status, 4 subjects are missing (2 females, 2 males/ 4 CRPS type 1).

^f^Due to unavailable information of level of education, 15 subjects are missing (13 females, 2 males/ 15 CRPS type 1).

^g^A significant result is seen between female/male and upper secondary school/post-secondary education.

^h^Due to unavailable information of duration of symptoms for diagnosis, 13 subjects are missing (11 females, 2 males/ 8 CRPS type 1, 5 CRPS type 2.

^i^Due to unavailable information of CRPS in the dominant hand, 42 subjects are missing (26 females, 16 males / 34 CRPS type 1, 8 CRPS type 2)

The frequency of smokers in the entire study population (34%) was significantly higher (p < 0.001) compared to a reference population (12%) from the Region of Skåne (men and women, 18-80 years of age, 2008-2020 [37]). The proportion of the study population living in a single household (32%) was not statistically different (p = 0.30) from the reference population (27%) [38]. The level of education (based on the grouping of elementary school, upper secondary school, and post-secondary school) in the study population was distributed as 6%, 67%, and 27% respectively. The corresponding figures in the reference population (age 25-64 and 2011-2020 in the Region of Skåne) were 12%, 42% and 43% respectively [39]. A significant difference was seen when comparing the groups concerning elementary school and upper secondary school (p = 0.008) as well as when comparing upper secondary school with post-secondary education (p < 0.001). However, no statistically significant difference was seen when comparing elementary school with post-secondary education. When analysing the period from the onset of initial symptoms until diagnosis, the time lag in type 2 subjects was significantly longer than that observed in type 1 subjects (p = 0.012). The dominant hand had no impact on the presence of CRPS.

**Fig 2:**
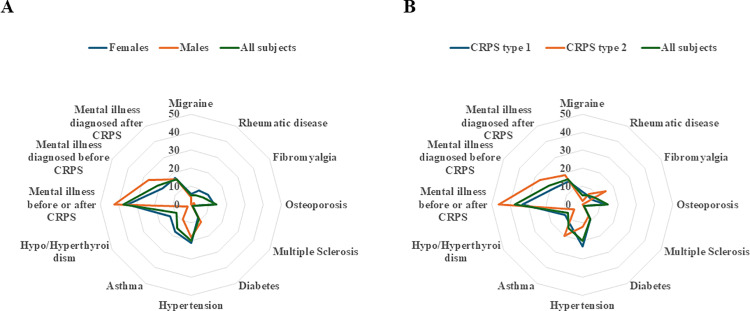
Spider diagram showing the comorbidities of the subjects with Complex Regional Pain Syndrome (CRPS) in the upper limb divided by sex (a) and type of CRPS (b). Please see S1 Table for detailed percentages and p-values.

Addressing co-morbidities as potentially influencing the type of CRPS, osteoporosis was significantly more frequent among subjects with CRPS type 1 as compared to subjects diagnosed with CRPS type 2 (p = 0.01). However, no other significant differences between sex or CRPS types regarding other co-morbidities could be identified (Fig 2a and 2b; S1 Table). In the entire study population, 37% had a mental illness, as defined in Methods (i.e., treated depression, anxiety, or PTSD), of whom 21% got such a diagnosis before the onset of CRPS and 16% after, giving a 76% increase of mental illnesses after receiving diagnosis with CRPS. The proportion of the present subjects, who had a mental illness at any time, was significantly higher (p < 0.001) than found in the reference group (14%; including subjects stating a reduced mental well-being, at the age of 16-84 and 2008-2016 [40]). Comparing the proportion of the study group who had mental illness before diagnosis with CRPS (21%), no significant difference was found (p = 0.91) compared to the reference group.

### Impact of previous surgery and history of pain on subjects diagnosed with CRPS

There was a significant difference between subjects diagnosed with CRPS type 1 and CRPS type 2 regarding previous surgery in the same hand as the current CRPS-diagnosed limb. A higher proportion of the subjects diagnosed with CRPS type 2 had undergone surgery on the same hand compared to subjects diagnosed with CRPS type 1 (p = 0.003). No other statistically significant differences between sex or between CRPS type regarding previous surgery, pain disorders, or previous contact with a pain management clinic were noted (S2 Table).

### Triggers and symptoms

**Fig 3:**
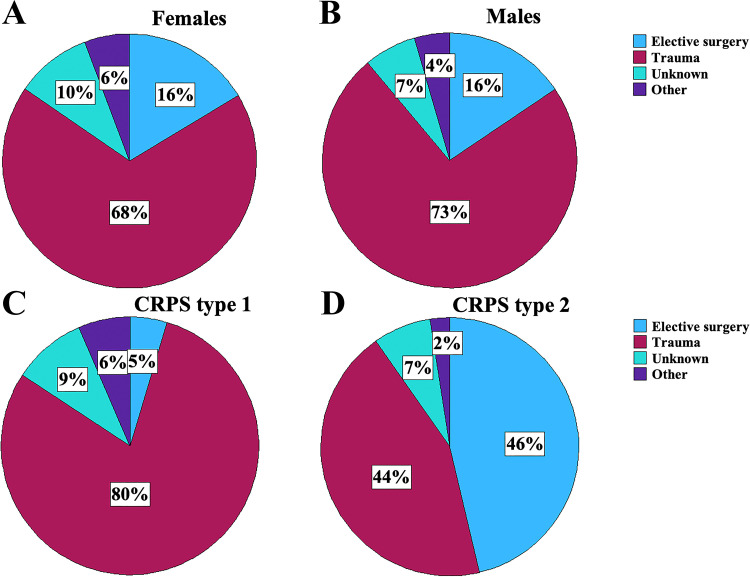
Pie chart showing the triggers for subjects with Complex Regional Pain Syndrome (CRPS) in the upper limb divided by females (a), males (b), CRPS type 1 (c), and CRPS type 2 (d).

**Fig 4:**
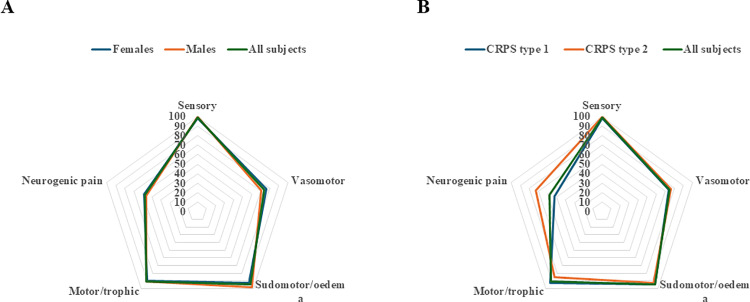
Spider diagrams showing the symptoms of the subjects with Complex Regional Pain Syndrome (CRPS) in the upper limb divided by sex (a) and type of CRPS (b).

Triggers and symptoms related to sex and type of CRPS are described in Figs 3 and 4, respectively. A significant difference between subjects diagnosed with CRPS type 1 and CRPS type 2 regarding triggers was observed (p < 0.001; Fig 3). Subjects diagnosed with CRPS type 1 had a higher proportion of physical trauma as a trigger compared to subjects diagnosed with CRPS type 2, who in contrast had elective surgery as a more common trigger. Sensory symptoms as well as sudomotor symptoms/oedema were seen in almost all subjects regardless of group. There were almost no differences between men and women regarding symptoms (Fig 4a). The only major difference between CRPS type 1 and CRPS 2 was the frequency of neuropathic pain (Fig 4b). Among subjects diagnosed with CRPS type 2, neuropathic pain was significantly more frequent than in type 1 subjects (p = 0.02).

### Clinical investigations and treatments

Clinical investigations and treatments are presented in S3 Table. The method used for diagnosis, X-ray, and neurophysiology, differed significantly between subjects diagnosed as having CRPS type 1 and CRPS type 2 (p < 0.001 and p = 0.003, respectively; S3 Table). Hence, it was more likely that subjects diagnosed with CRPS type 1 had been investigated using X-ray. Furthermore, it was more likely that subjects diagnosed with CRPS type 2 had been assessed by neurophysiological methods as compared to subjects diagnosed with type 1. However, no significant differences between sex or between CRPS types were found as regards recommended treatments for CRPS (i.e., vitamin C, cortisone, cortisone injection, bisphosphonates, acetylcysteine, rehabilitation, or mirror therapy). In contrast, for other types of treatments (such as botulinum toxin injections, spinal cord stimulation, acupuncture, cognitive behavioural therapy, and amputation), a significant difference between subjects diagnosed with CRPS type 1 and CRPS type 2 were observed (p = 0.04). Hence, subjects diagnosed with CRPS type 2 were more likely to receive these types of treatments as compared to CRPS type 1 subjects. In addition, a significant difference between subjects diagnosed with CRPS type 1 and CRPS type 2 regarding analgesic treatment with local anaesthesia (such as patches with capsaicin and lidocaine) was observed as subjects diagnosed with CRPS type 2 were more likely to receive local anaesthesia compared to subjects diagnosed with CRPS type 1 (p < 0.001). No other statistically significant differences between women and men or between subjects diagnosed with CRPS type 1 and 2 regarding investigations or treatments were found.

### Outcome variables

The different variables concerning outcome are presented in Table 2. Subjects diagnosed with CRPS type 2 had surgery significantly more frequently compared with subjects diagnosed with CRPS type 1 (p < 0.001). In contrast, subjects diagnosed with CRPS type 1 were more often treated at the Department of Orthopaedic (p < 0.001). In contrast, CRPS type 2 subjects were more often treated at the Department of Hand Surgery (p = 0.03). Regarding return-visits to physicians, subjects with CRPS type 2 were found to have significantly more such visits compared to subjects with CRPS type 1 (p = 0.004) and women had significantly more return visits to a rehabilitation unit than men (p = 0.03). Subjects diagnosed with CRPS type 2 had a significantly longer follow-up time (p = 0.003). No other differences regarding the outcome variables were found (Table 2).

**Table 2:** Surgeries, follow-up, and outcome variables in subjects diagnosed with Complex Regional Pain Syndrome (CRPS) in the cohort and split by sex and type of CRPS.

	Study population (n = 149)	Female (n = 104)	Male (n = 45)	P-value	CRPS type 1 (n = 108)	CRPS type 2 (n = 41)	P-value
**Number of surgeries** ^a^	1 [0-2]	1 [0-2]	0 [0-2]	0.49	0 [0-1]	1 [1–3]	** < 0.001**
**Treating department (yes/no)**							
Hand surgery	80/69 (54/46)	60/44 (58/42)	20/25 (44/56)	0.14	52/56 (48/52)	28/13 (68/32)	**0.03**
Orthopedic	104/45 (70/30)	68/36 (65/35)	36/9 (80/20)	0.07	84/24 (78/22)	20/21 (49/51)	**<0.001**
Pain management clinic	90/59 (60/40)	61/43 (59/41)	29/16 (64/36)	0.51	60/48 (56/44)	30/11 (73/27)	0.06
**Return visits**							
Physician (numbers)	15 [8-27]	15 [8-26]	14 [8-29]	0.77	12 [7–23]	21 [12-32]	**0.004**
Rehabilitation unit (numbers)	15 [8-26]	17 [9-26]	11 [6–21]	0.03	15 [8–23]	17 [5-31]	0.73
**Follow-up (months)**	21 [8-43]	22 [9-43]	18 [6-46]	0.66	15 [7-36]	31 [15-62]	**0.003**
**Time of sick leave (months 100%)** ^b^				0.35			0.41
0-3 months	31 (25)	24 (28)	7 (18)	–	23 (26)	8 (22)	–
4-6 months	16 (13)	8 (9)	8 (21)	–	12 (14)	4 (11)	–
7-9 months	16 (13)	12 (14)	4 (10)	–	13 (15)	3 (8)	–
10-12 months	5 (4)	4 (5)	1 (2)	–	2 (2)	3 (8)	–
>12 months	56 (45)	37 (44)	19 (49)	–	37 (43)	19 (51)	–
**Back to work** ^c^				0.72			0.50
Yes	57 (50)	41 (53)	16 (44)	–	41 (51)	16 (47)	–
No	25 (22)	16 (20)	9 (25)	–	19 (24)	6 (18)	–
Partially	32 (28)	21 (27)	11 (31)	–	20 (25)	12 (35)	–
**Permanent incapable of work (yes/no)** ^d^	23/90 (20/80)	13/64 (17/83)	10/26 (28/72)	0.18	15/63 (19/81)	8/27 (23/77)	0.66

Values are presented as number and proportion of observations (n (%)) or median (interquartile range; IQR]. P-values are based on Chi-squared test (or Fisher´s exact probability test if a group had n <  5) for categorical variables or Mann-Whitney U-test for numerical data. A p-value of < 0.05 was considered statistically significant and is indicated in bold.

^a^Including any surgery that triggered the CRPS.

^b^Due to the large group of subjects who receive extended sick leave via outpatient care, after they have been discharged from hospital clinic, the time for sick leave has been calculated up to 12 months and therefore a division into groups has been made. Only subjects who worked before diagnosis have been included (124 subjects).

^c^Due to unavailable information about if subject is back to work, 35 subjects are missing (26 females, 9 males/ 28 CRPS type 1, 7 CRPS type 2).

^d^Due to unavailable information if subject is permanently incapable of work, 36 patients are missing (27 females, 9 males/30 CRPS type 1, 6 CRPS type 2)

### Linear regression analysis of outcome as follow-up time

The regression analysis did not show any association between the variables sex, smoking, previous surgery in the same hand, or neuropathic pain as symptom and follow-up time; the latter being used as a proxy for outcome. However, a significant association between the type of CRPS and the follow-up time (unstandardized B-coefficient value 17.4, CI 95% [5.2-29.6], p = 0.005; adjusted for age and sex) was found, indicating that a diagnosis of CRPS type 2, instead of type 1, increased the follow-up time with more than 17 months. Furthermore, a significant association between age at diagnosis and follow-up time (months) was detected (unstandardized B-coefficient value -0.7, CI 95% [-1.1 - -0.4], p < 0.001; adjusted for sex), which implies that for every year older a subject is when seeking care, the follow-up time will be reduced by approximately 3 weeks. The analysis also showed a significant association between the duration of symptoms until diagnosis and follow-up time (unstandardized B-coefficient value 0.3, CI 95% [0.2-0.4], p < 0.001; adjusted for age and sex), meaning that for each week following the start of symptoms before diagnosis, the follow-up time will increase with approximately 10 days.

## Discussion

Our study shows that subjects with earlier CRPS were mainly manual workers and a large proportion were smokers and had a lower grade of education. CRPS 2 resulted in a worse outcome at long-time follow-up and previous pain disorder and/or earlier contact with a pain management clinic was relatively common. Mental illness was not overrepresented before the CRPS diagnosis but increased notably following the diagnosis. As much as 45% were still on sick leave 12 months after the onset of CRPS and 20% were judged incapable of ever working again. The present study focuses on associated factors, triggers, and outcome, using different variables as proxies.

Women have a higher occurrence of CRPS than men with a ratio of 70/30, which is in concordance with the present and earlier studies [1,7,8,11,29,33,35]. This may be due to the frequency of trauma as the trigger, such as distal radius fractures and osteoporosis [41], which was observed in CRPS type 1. Significant, although not large, sex differences for age at initial diagnosis as well as for the type of CRPS were found, where the present ages for men and women at diagnosis agree with a previous study [21]. Interestingly, the entire study population had high BMI and were classified as overweight. BMI is earlier reported not to be an influencing factor for the development of CRPS [29]. The larger proportion of the study population being smokers compared to the reference population also indicates smoking as a potentially associated, or confounding, factor [35,42] in contrast to other studies [29]. Considering occupation, a clear majority had manual work as their profession. Thus, one may speculate if the manual work itself constitutes a risk factor for developing CRPS or whether the type of profession may affect the extent of health care needed, e.g., after trauma.

Previous studies have pointed to different co-morbidities, such as migraine, fibromyalgia, asthma, rheumatoid arthritis, mental illness as well as psychosocial factors, as potential risk or associated factors for CRPS [1,2,7,8,29,35,43]. Migraine, fibromyalgia, asthma, and rheumatoid arthritis were also present, although to a relatively low frequency among the subjects. Mental illness was more prominent as a disease among the subjects both before and after the diagnosis of CRPS. Treatment of mental illness is an important part of the management of patients with CRPS [44]. In the present study, mental illness, as defined, was the only diagnosis overrepresented in the study population compared to a reference population, but only if counting the total amount of subjects diagnosed with mental illness before and after diagnosis with CRPS; however, an increase in number of subjects after the diagnosis was observed.

Few studies have compared CRPS type 1 and CRPS type 2 and discriminated between them when describing triggers [3,7,11,33,45]. The most frequent present triggers, irrespective of sex, were trauma and elective surgery related to CRPS type 1 and CRPS type 2, respectively [7]. Some previous studies include CTS as a trigger for CRPS type 1 as long as no known nerve damage is detected during surgery, which in such cases would have resulted in CRPS type 2 diagnosis [8]. We defined all subjects who had elective surgery triggered by CTS or other nerve entrapments as having CRPS type 2 since CTS *per se* is a nerve injury and therefore should be stated as CRPS type 2 [6,15]. However, consistently classifying patients with CTS as CRPS type 2 will probably affect the significance of the difference when comparing CRPS types and triggers. An expected CRPS type 2 demonstrated neuropathic pain as one of the onset symptoms.

There was no difference in choice of treatment between sexes or types of CRPS regarding most treatment options. Surprisingly, a larger proportion of the subjects, irrespective of sex or type of CRPS, was treated with opioids, which should be considered in view of the opioid pandemic. A multidisciplinary treatment, with a biopsychosocial approach, is important in the treatment of CRPS [3,9]. Subjects with CRPS type 2 had twice as long follow-up time, used as a proxy for outcome, and almost twice as many return visits to physicians, indicating a more severe and difficult disease course for CRPS type 2; thus, both the physicians and the subjects maybe being more prone to try more invasive or extensive therapies. Overall, only 21% received mirror therapy despite previous studies reporting the rehabilitation benefits of such treatment [7,46].

CRPS type 1 subjects were treated mostly at the Department of Orthopaedic, while CRPS Type 2 subjects were treated more frequently at the Department of Hand Surgery due to the nature of the trigger and severity of CRPS. A nerve injury in CRPS Type 2 requires longer follow-up times and more frequent physician visits as it is difficult to treat and may severely impair hand function [47]. Linear regression analysis indicates a more severe course of the disease among CRPS type 2 subjects. Early recognition of symptoms and onset of treatment are considered crucial.

A limitation of the present study is the relatively small number of subjects, which, however, is not different from other studies of CRPS in the upper limb. We included only subjects at long-term follow-up and with CRPS in the upper limb as we wanted to study specific triggers for CRPS in the upper limb and outcome over time. Another limitation of the present study is that we did not include subjects from primary care, and only from the specialist healthcare, as we did not have access to such data. The assumption was made that diagnosed subjects in primary care are probably referred to and assessed in the specialist healthcare. This may potentially influence the number of found subjects as well as follow-up time, time of sick leave, and numbers of return visits to physicians and rehabilitation units. Many patients, especially those living far from specialist clinics, may more likely have been treated at the rehabilitation units at the local primary care. All subjects, defined as having CRPS in the upper limb, were diagnosed and treated in the Region of Skåne giving rise to a well-defined study group, which is a strength, despite that we did not include a “control group”; i.e., subjects that were diagnosed with an upper limb traumatic or elective diagnosis. However, we related our data to a reference population. In addition, all patient folders were carefully reviewed and agreed upon by two independent researchers (AP and EL), and at times of uncertainty, the senior authors were consulted (LD and EN). Through a careful screening of the information among all subjects with a suspected CRPS diagnosis in Region of Skåne, subjects with the correct symptoms, but without the appropriate ICD-code, have been included, which is a strength. There are no specific biomarkers to diagnose CRPS, but a spectrum of biomarkers might be useful in the future to predict the diagnosis, and guide treatment and prognosis [23].

We conclude that smoking as well as a low level of education may be considered as potential associated factors for CRPS, which may indicate that socioeconomic factors play a role. Not only physical aspects should be prioritized in the treatment of CRPS, but also mental health. Subjects who develop CRPS, frequently with trauma as a trigger, and especially those with a previous nerve injury, risk severe long-term symptoms. Long sick leave and a high proportion of subjects who never return to work need attention from health care personnel working with subjects with CRPS.

## Supporting information

S1 Table Comorbidity and mental illness before and after diagnosis among subjects with Complex Regional Pain Syndrome (CRPS). (DOCX)

S2 Table Previous history of surgery in hand/arm and history of pain in subjects with Complex Regional Pain Syndrome (CRPS) in the entire population and split by sex and type of CRPS. (DOCX)

S3 Table Investigations and treatment options in subjects diagnosed with Complex Regional Pain Syndrome (CRPS) in the entire cohort and split by sex and type of CRPS. (DOCX)
