# Supplementary material for: Associated factors, triggers and long-term outcome in Complex Regional Pain Syndrome (CRPS) in the upper limb – A descriptive cross-sectional study
Source: PLoS One. 2025 Mar 28;20(3):e0320263. doi: 10.1371/journal.pone.0320263 (PMC11952230; doi:10.1371/journal.pone.0320263)
Supplement: S1 Table — (DOCX) [file pone.0320263.s001.docx]

**Supplemental Table S1. Comorbidity and mental illness before and after diagnosis among subjects with Complex Regional Pain Syndrome (CRPS)**

|  | Study population  (n = 149) | Female  (n = 104) | Male  (n = 45) | P-value | CRPS type 1  (n = 108) | CRPS type 2  (n = 41) | P-value |
| --- | --- | --- | --- | --- | --- | --- | --- |
| Type of comorbidity  (yes /no) |  |  |  |  |  |  |  |
| Migraine | 8/141  (5/95) | 6/98  (6/94) | 2/43  (4/96) | 1.0 | 7/101 (7/93) | 1/40  (2/98) | 0.45 |
| Rheumatic disease | 9/140  (6/94) | 9/95  (9/91) | 0/45  (0/100) | 0.06 | 6/102 (6/94) | 3/38  (7/93) | 0.71 |
| Fibromyalgia | 12/137  (8/92) | 11/93  (11/89) | 1/44  (2/98) | 0.11 | 6/102  (6/94) | 6/35  (15/85) | 0.07 |
| Osteoporosis | 14/135  (9/91) | 13/91  (13/87) | 1/44  (2/98) | 0.07 | 14/94 (13/87) | 0/41 (0/100) | **0.01** |
| Multiple Sclerosis | 1/148  (1/99) | 1/103  (1/99) | 0/45  (0/100) | 1.00 | 1/107  (1/99) | 0/41  (0/100) | 1.00 |
| Diabetes | 13/136  (9/91) | 8/96  (8/92) | 5/40  (11/89) | 0.53 | 10/98 (9/91) | 3/38  (7/93) | 1.00 |
| Hypertension | 30/119  (20/80) | 22/82 (21/79) | 8/37  (18/82) | 0.82 | 25/83 (23/77) | 5/36 (12/88) | 0.17 |
| Asthma | 22/127  (15/85) | 18/86 (17/83) | 4/41  (9/91) | 0.18 | 14/94 (13/87) | 8/33 (20/81) | 0.31 |
| Hypo/Hyper-  thyroidism | 14/135  (9/91) | 13/91 (13/88) | 1/44  (2/98) | 0.07 | 12/96 (11/89) | 2/39  (5/95) | 0.35 |
| Mental illness *before or after* CRPS | 55/94  (37/63) | 36/68 (35/65) | 19/26 (42/58) | 0.38 | 36/72 (33/67) | 19/22 (46/54) | 0.14 |
| Mental illness diagnosed *before* CRPS diagnosis | 31/118  (21/79) | 19/85  (18/82) | 12/33  (27/73) | 0.25 | 20/88  (18/82) | 11/30 (27/73) | 0.26 |
| Mental illness diagnoses *after* CRPS diagnosis | 24/122  (16/84) | 17/85  (17/83) | 7/37  (16/84) | 0.91 | 16/89  (15/84) | 8/33  (19/81) | 0.53 |

Values are presented as number and proportion of observations (%). Mental illness was based on the information in the patient folders about treated conditions about their mental condition, like depression, anxiety, and PTSD, but no severe psychiatric conditions were present (e.g., schizophrenia).
