# Supplementary material for: Associated factors, triggers and long-term outcome in Complex Regional Pain Syndrome (CRPS) in the upper limb – A descriptive cross-sectional study
Source: PLoS One. 2025 Mar 28;20(3):e0320263. doi: 10.1371/journal.pone.0320263 (PMC11952230; doi:10.1371/journal.pone.0320263)
Supplement: S2 Table — (DOCX) [file pone.0320263.s002.docx]

**Supplemental Table S2. Previous history of surgery in hand/arm and history of pain in subjects with Complex Regional Pain Syndrome (CRPS) in the entire population and split by sex and type of CRPS**

|  | **Entire study**  **population**  (n = 149) | **Female**  (n = 104) | **Male**  (n = 45) | **P-value** | **CRPS type 1**  (n = 108) | **CRPS type 2**  (n=41) | **P-value** |
| --- | --- | --- | --- | --- | --- | --- | --- |
| **Previous surgery^a^** |  |  |  |  |  |  |  |
| Same hand as CRPS (yes/no) | 25/123  (17/83) | 16/88 (15/85) | 9/35 (20/80) | 0.45 | 12/95 (11/89) | 13/28 (32/68) | **0.003** |
| Other hand as CRPS (yes/no) | 9/139  (6/97) | 6/98  (6/94) | 3/41 (7/93) | 1.0 | 6/101 (6/94) | 3/38 (7/93) | 0.71 |
| **Previous pain disorder (yes/no)** | 48/101  (32/68) | 36/68 (35/65) | 12/33 (27/73) | 0.34 | 32/76 (30/70) | 16/25 (39/61) | 0.27 |
| **Previous diagnosed with CRPS^b^ (yes/no)** | 6/143  (4/96) | 4/100 (4/96) | 2/43 (4/96) | 1.00 | 5/103 (5/95) | 1/40 (2/98) | 1.00 |
| **Previous contact with pain management clinic (yes/no)** | 11/138  (7/93) | 6/98  (6/94) | 5/40 (11/89) | 0.31 | 8/100 (7/93) | 3/38 (7/93) | 1.00 |

Values are presented as number and proportion of observations (n (%)). P-values are based on Chi-squared test (or Fisher´s exact probability test if a group had n < 5) for categorical variables. A p-value of <0.05 was considered as statistically significant and is indicated in bold.

Due to unavailable information of previous surgery 1 subject is missing (1 male / 1 CRPS type 1).

^b^ Subject has previously been diagnosed with CRPS in any limb.
