# Supplementary material for: Associated factors, triggers and long-term outcome in Complex Regional Pain Syndrome (CRPS) in the upper limb – A descriptive cross-sectional study
Source: PLoS One. 2025 Mar 28;20(3):e0320263. doi: 10.1371/journal.pone.0320263 (PMC11952230; doi:10.1371/journal.pone.0320263)
Supplement: S3 Table — (DOCX) [file pone.0320263.s003.docx]

**Supplemental Table S3. Investigations and treatment options in subjects diagnosed with Complex Regional Pain Syndrome (CRPS) in the entire cohort and split by sex and type of CRPS**

|  | **Entire study population**  (n = 149) | **Female**  (n = 104) | **Male**  (n = 45) | **P-value** | **CRPS type 1**  (n = 108) | **CRPS type 2**  (n = 41) | **P-value** |
| --- | --- | --- | --- | --- | --- | --- | --- |
| **Investigation (yes/no)** |  |  |  |  |  |  |  |
| X-ray, hand | 111/38  (75/25) | 79/25 (76/24) | 32/13 (71/29) | 0.53 | 92/16 (85/15) | 19/22 (46/54) | **< 0.001** |
| CT/MRI^a^ hand | 56/93  (38/62) | 38/66 (37/63) | 18/27 (40/60) | 0.69 | 41/67 (38/62) | 15/26 (37/63) | 0.88 |
| CT/MRI neck or shoulder | 36/113  (24/76) | 25/79 (24/76) | 11/34 (24/76) | 0.96 | 24/84 (22/78) | 12/29 (29/71) | 0.37 |
| Neurophysiology | 71/78  (48/52) | 50/54 (48/52) | 21/24 (47/53) | 0.87 | 43/65 (40/60) | 28/13 (68/32) | **0.003** |
| **Treatments (yes/no)** |  |  |  |  |  |  |  |
| Vitamin C | 53/96  (36/64) | 38/68 (35/65) | 17/28 (38/62) | 0.71 | 41/67 (38/62) | 12/29 (29/71) | 0.32 |
| Corticosteroids | 51/98  (34/66) | 33/71 (32/68) | 18/27 (40/60) | 0.33 | 38/70 (35/65) | 13/28 (32/68) | 0.69 |
| Cortisone injection | 22/127  (15/85) | 17/87 (16/84) | 5/40 (11/89) | 0.41 | 18/90 (17/83) | 4/37 (10/90) | 0.29 |
| Bisphosphonate | 16/133  (11/89) | 10/94 (10/90) | 6/39 (13/87) | 0.57 | 10/98 (9/91) | 6/35 (15/85) | 0.38 |
| Acetylcysteine | 11/138  (7/93) | 5/99  (5/95) | 6/39 (13/87) | 0.09 | 8/100 (7/93) | 3/38 (7/97) | 1.00 |
| Treatment at rehabilitation unit | 146/3  (98/2) | 101/3 (97/3) | 45/0 (100/0) | 0.55 | 105/3  (97/3) | 41/0 (100/0) | 0.56 |
| Mirror Therapy | 31/118  (21/79) | 21/83 (20/80) | 10/35 (22/78) | 0.78 | 26/82 (24/79) | 5/36 (12/88) | 0.11 |
| Others^b^ | 16/133  (11/89) | 12/92 (11/89) | 4/41 (9/91) | 0.78 | 8/100 (7/93) | 8/33 (19/81) | **0.04** |
| **Analgesics (yes/no)** |  |  |  |  |  |  |  |
| Paracetamol | 119/30  (80/20) | 85/19 (82/18) | 34/11 (76/24) | 0.39 | 89/19 (82/18) | 30/11 (73/27) | 0.21 |
| NSAID^c^ | 65/84  (44/56) | 44/60 (42/58) | 21/24 (47/53) | 0.62 | 50/58 (46/54) | 15/26 (37/63) | 0.29 |
| Opioids | 117/32  (79/21) | 78/26 (75/25) | 39/6 (87/13) | 0.11 | 84/24 (78/22) | 33/8 (80/20) | 0.72 |
| Local anesthesia^d^ | 20/129  (13/87) | 12/92 (12/88) | 8/37 (18/82) | 0.31 | 7/101 (7/93) | 13/28 (32/68) | **< 0.001** |
| Anticonvulsants | 86/63  (58/42) | 58/46 (56/44) | 28/17 (62/38) | 0.46 | 60/48 (56/44) | 26/15 (63/37) | 0.39 |
| Anti-depressants/anxiety | 33/115  (22/78) | 21/82 (20/80) | 12/33 (27/73) | 0.40 | 22/85 (21/79) | 11/30 (27/73) | 0.41 |

Values are presented as number and proportion of observations (n (%)) or median (interquartile range; IQR]. P-values are based on Chi-squared test (or Fisher´s exact probability test if a group had n < 5) for categorical variables or Mann-Whitney U-test for numerical data. A p-value of <0.05 was considered as statistically significant and is indicated in bold.

Computed tomography (CT) / Magnetic Resonance Imaging (MRI)

^b^ Example of other treatments may be botulinum toxin injection, spinal cord stimulation, acupuncture, cognitive behavioral therapy, and amputation.

^c^ Non-steroidal anti-inflammatory drug (NSAID).

^d^ Local anaesthesia, such as patches with capsaicin and lidocaine.
